# Supplementary material for: Prevalence of malaria and associated factors among symptomatic pregnant women attending antenatal care at three health centers in north-west Ethiopia
Source: PLoS One. 2022 Apr 7;17(4):e0266477. doi: 10.1371/journal.pone.0266477 (PMC8989222; doi:10.1371/journal.pone.0266477)
Supplement: S1 File — (ZIP) [file pone.0266477.s001.zip › Supporting information files/questionnaire.docx]

***Questionnaire (English version)***

Bahirdar University, College of Medicine and Health Sciences, School of Health Sciences, Department of Medical Laboratory Science

Questionnaire to collect data on socio-demographic characteristics of participants and factors associated with malaria among symptomatic pregnant women at Tis Abay, Zenzelma and Hamusit Health centers, North west Ethiopia

**Participant Identification**

Facility name ________________ Year __________ Participant code ________

Participant’s address: (Sub city/Keble) ________ Telephone **____________**

Data collector’s name_________________________date____________signature__________

1. Age: ˂20 20-24 25-29 30-34 35-49 40-44 ≥45

2. Residence: Rural Urban

3. Educational status: can’t read and write primary school secondary school

Higher education

4. Occupation: farmer government employee house wife others (specify) --------------

5. Gestational age / trimester of pregnancy/

First trimester second trimester third trimester

6. Gravidity: primigravidae secundigravidae multigravidae

7. How long is distance from your home to health center? ≤ 1 hr 1- 2hrs >2hrs

8. Do you use bed nets? Yes No

If NO, Why? Not available Not comfortable others (specify) -----------------------------------

9. Please mark if there are the following conditions around your residence / home/

Marsh area stagnant water left over materials for tire irrigation

10. Do you spend at the evening/dusk/ by performing different activities outside home?

Yes No

11. Do you sleep at outdoors at night? Yes No

12. Signs and symptoms during arrival to health center

Fever Joint pain Headache Dizziness

Vomiting Convulsion Malaise Fatigue

Jaundice Anemia others (specify) ----------------

13. Other underlying chronic diseases

Diabetes Mellitus Tuberculosis HIV/ AIDS Others (specify) ----------------

Result ------------------------

| *Plasmodium* species | Stage |
| --- | --- |
|  |  |
|  |  |

III. Questionnaire in Amharic version

ባህርዳር ዩኒቨርሲቲ፣የህክምናና ጤና ሳይንስ ኮሌጅ፣ የጤና ሳይንስ ት/ቤት፣ ህክምና ላቦራቶሪ ት/ት ክፍል

የወባ በሽታ ምልክት በሚያሳዩ ና ጢስ አባይ፣ ዘንዘልማ ና ሐሙሲት ጤና ጣቢያዎች ላይ የእርግዝና ክትትል በሚያደርጉ ነፍሰጡር እናቶች ላይ ያለዉን የወባ በሽታ ስርጭትና ተዛማጅ ምክንያቶችን ለማጥናት መረጃ ለመሰብሰብ የተዘጋጀ ቃለመጠይቅ

የጤና ተቁዋሙ ስም----------------------ዓ.ም----------------የጥናቱ ተሳታፊ መለያ ቁጥር--------

መረጃዉን የወሰደው ግለሰብ ስም---------------------------------ቀን------------------ፊርማ------

እባክዎ ለጥናቱ መሳካት ያግዘን ዘንድ ጥያቄዎችን በጥንቃቄ እንዲመልሱልን በትህትና እንጠይቃለን፡፡

1. ዕድሜ : --------------

2. መኖሪያ:
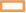
 ከተማ
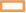
 ገጠር

3. የትምህርት ደረጃ፡


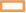
 ያልተማሩ
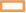
 1ኛ ደረጃ
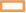
 2ኛ ደረጃ
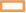
 ከፍተኛ ደረጃ

4. ሙያ፡
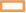
 አ/አደር
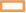
 መ/ሠራተኛ
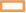
 የቤት እመቤት
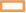
 ሌላ--------

5. ወራተ ጽንስ


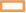
 1ኛዉ ሲሶ ወሰን
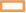
 2ኛዉ ሲሶ ወሰን
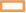
 3ኛዉ ሲሶ ወሰን

6. የእርግዝና ጊዜ፡
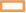
 የመጀመሪያ
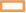
 ሁለተኛ ጊዜ
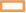
 ብዙ ጊዜ

7. ከቤትዎ እስከ ጤና ጣቢያዉ ያለዉ ርቀት በሰአት ፡
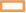
 ≤ 1ሰአት
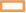
 1-2 ሰአት
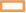
 >2ሰአት

8. አጎበር ይጠቀማሉ?
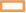
 አዎ
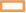
 አልጠቀምም

ማይጠቀሙ ከሆነ ለምን ?
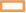
 የለኝም
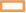
 አይመችም
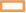
 ሌላ-------

9. መኖሪያ አካባቢዎ የሚከተሉት ነገሮች ካሉ ምልክት ያርጉ


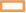
 ረግረጋማ ቦታ
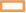
 የቆመ ዉሃ
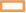
 መስኖ
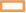
 የወዳደቁ ብረቶች፣ መኪና ጎማ

10. ምሽት ላይ የተለያዩ ተግባራትን በማከናወን ከቤት ዉጭ ያሳልፋሉ?


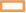
 አዎ
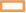
 የለም

11. ሌሊት ሚተኙት ከቤትዎ ዉጭ ነዉ?
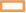
 አዎ
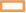
 አይደለም

12. ጤና ጣቢያ ሲደርሱ ሚያሳዩት ምልክቶች፡


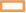
 ትኩሳት
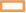
 ቁርጥማት
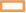
 የራስ ምታት
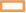
 ራስን ማዞር
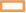
 ትዉኪያ
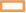
 መንቀጥቀጥ
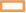
 መጫጫን/ ጭንቀጥ
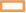
 ድካም
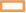
 ሌላ---------

13. ሌሎች ተጉዋዳኝና የቆዩ በሽታዎች


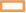
 የስኩዋር በሽታ
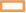
 ቲቢ
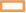
 ኤች አይ ቪ/ ኤድስ
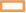
 ሌላ---------
