# Supplementary material for: Prevalence of malaria and associated factors among symptomatic pregnant women attending antenatal care at three health centers in north-west Ethiopia
Source: PLoS One. 2022 Apr 7;17(4):e0266477. doi: 10.1371/journal.pone.0266477 (PMC8989222; doi:10.1371/journal.pone.0266477)
Supplement: Attachment — Submitted filename: Response to reviewers.docx [file pone.0266477.s003.docx]

**Point by point response to reviewer#2 comments**

First of all, we authors would like to thank reviewer#2` again for comments to make this manuscript more plausible. As much as possible, we authors would like to address your comments here.

Minor comments

1. Line 48: the paper shows that malaria is A significant problem, not THE significant problem.

**Response**: well taken; dear reviewer, we have corrected as you suggested. “Malaria is the disease…”

2. I still contend that lines 63-71 are text book descriptions of malaria which do not belong in this article. Reviewer 1 suggests also that some of this text is redundant.

**Response:** well taken; dear reviewer, this section is about the transmission and life cycle in human host. Actually, it is in lines 49-55 from revised manuscript. We understood that the idea was… “Malaria is transmitted to humans by the bite of female *Anopheles* mosquitoes. *Anopheles* mosquitoes feed on blood for survival and production of eggs. During feeding, infected female *Anopheles* mosquito inoculates the infective sporozoites from its salivary gland into human skin. After inoculation, parasites circulate with blood and those reaching to the liver undergo one cycle development. Then, parasites infect and multiply inside red blood cells to bring the characteristic signs and symptoms. The clinical manifestations include fever, joint pain, chills, headache and vomiting which usually appear between 10 and 15 days after the Anopheles mosquito bite. In addition, malaria may also be transmitted through blood transfusion and congenitally”. Even we haven’t got redundancy here. **We hope we have got your idea. Thank you!**

3. Line 136 Sample size: I still do not find this clear, and the authors’ written response is also unclear (response to reviewers, page 11).

**Response:** well taken; dear reviewer,

The sample size was determined using single population proportion formula based on 50% prevalence, 95% confidence level and 5% margin of error with 10% non-response rate as follows.

n = (Zα/2)2 P (1-P)

d2

n= (1.96)2 (0.5) (1–0.5)2 = 384 plus 10% non-response rate =422

(0.05)2

Where;

n = number of sample size.

P= the proportion (p = 0.5) taken as 50% prevalence

d= marginal error between the sample and population (0.05).

Z= critical value at 95% certainty (1.96), considering 10 % non-responsive rate.

But, according to information obtained from health centers, the total number of symptomatic pregnant women diagnosed in the three study areas in 2019/2020 was 1202 which is finite population or less than 10.000. So, according to statistics, adjustment (correction) formula is used to calculate sample sizes for population of less than 10*,*000. This is because a given sample size provides proportionately more information for small population than for a large population and we have reduced the sample size based on the following formula.

nf = n/(1+(n/N)) where, nf= final sample size i.e. sample size from finite population

n= sample size from an infinite population i.e. 422

N= total number of target population from all three health centers who visited in previous year i.e. 1202; So, nf= 422/1+ (422/1202)) = 312 (total number of symptomatic pregnant women participated in the study).

4. Line 185-6: The wording is not clear here. If the expert microscopist’s review was taken as final, please say so. Or describe who confirmed discordant results and how.

**Response:** Well taken**.** Dear reviewer, the review (re-examination) by microscopists in APHI was taken as final. Because, it is the APHI that assesses malaria quality assurance and gives final decision about species type in every health institutions around.

Thank you!
