# Supplementary material for: Prevalence of malaria and associated factors among symptomatic pregnant women attending antenatal care at three health centers in north-west Ethiopia
Source: PLoS One. 2022 Apr 7;17(4):e0266477. doi: 10.1371/journal.pone.0266477 (PMC8989222; doi:10.1371/journal.pone.0266477)
Supplement: Attachment — Submitted filename: Response to reviewers.docx [file pone.0266477.s004.docx]

**Point by point response to reviewers, comments**

**Authors’ Response to the reviewers, comments**

First, we authors would like to thank reviewers` for their comments to make this manuscript more plausible. As much as possible, we authors would like to address your comments here.

**Reviewer #1: Abstract**

**Background**

Line 18......There is no need of having this sentence since the previous sentence in line 15-17 has already explained the problem that prompted this study to be conducted.

**Response:** Well taken. Dear reviewer, we have removed it

**Results**

There should be no space between the numbers and the percentage symbol, but rather there should be a space between the percentage symbol and the brackets. The correction should be done throughout the document.

**Response:** Well taken. We have corrected

**Conclusion**

Lines 38-40.should state that...."malaria is still a public health problem among pregnant women" ....instead of ..."in the study area

**Response:** Well taken and we have corrected as you suggested

Line 42....change "are" to "were", "increase" to "increased/increasing" the prevalence........

**Response:** Well taken and changed

**Main body**

Line 46.....the sentence is left hanging, here it would be good to mention the five Plasmodium species infecting human.

**Response:** Well taken. We have included five species.

52.....Does the Anopheles inoculate the infective sporozoites into the skin?

**Response:** Well taken. Dear reviewer, it is to mean human circulation and we have corrected it.

Line 69-70...This sentence is not supposed to to be in that paragraph as it brings confusion. Whereas the previous sentence is on control measures, this sentence is on the risk factors for malaria infection in pregnant women.......thus there is no continuation.

**Response**: Well taken. We have removed it.

Line 133.....What is HCG? Spell it out.

**Response**: Well taken. Dear reviewer, it is to mean Human Chorionic Gonadotropin hormone and we have included it.

Line 176.....Remove the word "have".

**Response**: Well taken and we have removed it.

Table 2.....Remove the frequency column as the figures in this column have already presented in the next column.

**Response**: Well taken and we have removed.

Line 189...change "have" to "had". Do the same for line 191.

**Response**: Well taken. We have changed.

Line 193....change "are" to "were"

**Response:** Well taken. We have changed.

Line 200....."this study results"...should be changed to..."the prevalence of malaria in this study".

**Response:** Well taken and changed.

Line 201...."than study results"....to...."than the prevalence in Burkina Faso".

**Response**: Well taken and changed.

Line 212...."than the study results".....to..."than the prevalence in".

**Response**: Well taken and changed.

Line 233....."shown"...to..."showed"

**Response**: Well taken and changed

**Reviewer #2:** The authors have responded to my comments. I don't think textbook description of the malaria life cycle is a good use of space but will let this pass.

Response: Dear reviewer, we have received. Thank you!
